# The effect of presenting complaint on the risk of developing ventilator-associated pneumonia for patients intubated in an academic emergency department

**DOI:** 10.1186/2197-425X-3-S1-A99

**Published:** 2015-10-01

**Authors:** L DeLuca, T Durns, R Miller, J Yeaton, A Pickering, Z Roward, D Sabb, KR Denninghoff

**Affiliations:** Department of Emergency Medicine, University of Arizona, Tucson, United Statesx

## Introduction

Ventilator associated pneumonia (VAP) is a complication of mechanical ventilation that increased ICU length of stay and mortality. Eckert found 26% of trauma patients intubated in the ED develop VAP as compared to 6.5% of those intubated in the ICU. Green demonstrated that 70% of critically ill patients were intubated pre-hospital or in the ED and 18.2% remained in the ED for more than 4 hours.

## Objectives

To identify patients intubated in the ED who are at risk for VAP and characterize the effect of presenting complaint on VAP risk and prevalence.

## Methods

A retrospective study was performed using an existing QI database of patients intubated in the ED. For the purposes of this study. "At-risk for VAP" was defined as intubated >48 hours, with no significant abnormality on chest x-ray in the first 48 hours. “At-risk” patients were identified as VAP positive if they had a new persistent infiltrate on CXR with temperature outside 36°-38°C, and leukocyte count outside 4,000-12,000. Chart review was performed in order to categorize patients by presenting complaints and determine VAP risk and incidence.

## Results

**Figure 1:**
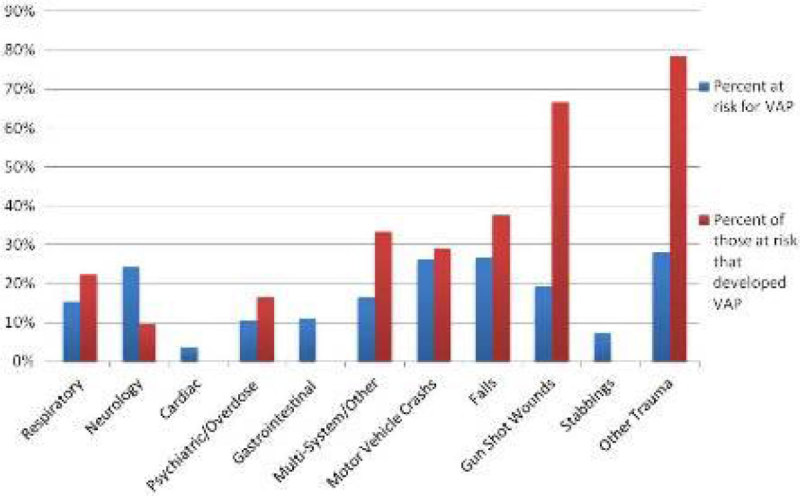
**VAP risk and Incidence by Presenting Complaint.**

539 patients were included in the ED intubation cohort, of which 244 presented with traumatic complaints. Within the group, 25% (60) were found to be at risk for VAP and 45% (27) of these developed VAP. 295 patients had medical presenting complaints. 16% (47) of medical patients were at risk for VAP and 17% (8) of these developed VAP. Trauma of unknown or less prevalent mechanism was categorized as Other Trauma, including TBI's and multi trauma. 28% of this group was at risk for VAP and 79% of these were VAP positive. 19% of gunshot wounds were at risk and 67% developed VAP. Neurology complaints presented with a low occurrence with 24% at risk and only 10% of these developing VAP. Cardiac, gastrointestinal and stabbing complaints had no incidences of VAP. Table 1 and 2, as well Figure 1 detail VAP risk and incidence by presenting complaints.

**Table 1:** Medical Patients VAP Risk and Incidence.

	Respiratory	Neurologic	Cardiac	Psychiatric	GI	Multisystem / other
Total Patients	59	87	28	57	9	55
% at risk for VAP	9 (15%)	21 (24%)	1 (4%)	6 (11%)	1 (11%)	9 (16%)
% at risk who developed VAP	2 (22%)	2 (10%)	0 (0%)	1 (17%)	0 (0%)	3 (33%)

**Table 2:** Trauma Patients VAP Risk and Incidence.

	MVA	Falls	GSW	Stabbing	Other Trauma
Total Patients	119	30	31	14	50
% at risk for VAP	31 (26%)	8 (27%)	6 (19%)	1 (7%)	14 (28%)
% at risk who developed VAP	9 (29%)	3 (38%)	4 (67%)	0 (0%)	11 (79%)

## Conclusions

Patients that arrived to the ED after a traumatic injury were at higher risk for VAP and developed VAP more frequently than those who presented with medical complaints. Future study, including a larger sample, is needed to continue to characterize VAP risk as related to presenting complaint and to determine interventions to reduce this risk.
